# Two variants of ‘constrained participation’ in the care of vulnerable adults: A proof-of-concept study

**DOI:** 10.1177/09697330231169930

**Published:** 2023-05-17

**Authors:** Kristján Kristjánsson, Kristín Thórarinsdóttir

**Affiliations:** 1724 University of Birmingham, Birmingham, UK; 64391 University of Akureyri, Akureyri, Iceland

**Keywords:** patient participation, constrained participation, fought-for participation, forced-to participation, vulnerable older adults

## Abstract

There has been a radical turn towards ideals of patient autonomy and person-centred care, and away from historically entrenched forms of medical paternalism, in the last 50 years of nursing practice. However, along the way, some shades of grey between the areas of ideal patient participation and of outright patient non-participation have been missed. The current article constitutes an exploratory proof-of-concept study of the real-world traction of a distinction-straddling concept of ‘constrained participation’ and its two sub-concepts of ‘fought-for participation’ and ‘forced-to participation’. In order to concretise these additions to the conceptual terrain of person-centred participation and its anti-theses, we apply them to themes in the care of vulnerable older adults. In the final section, we close by eliciting some characterological, educational and clinical implications of adding these new tools also to the conceptual repertoire of nursing practice and education.

## Introduction

Patient participation is regarded as an ideal in modern health care, and it has been strengthened in regulations and agendas at national and international levels.^1,2^ Its evolution harks back to existing socio-political movements in the 1960s, which emphasised the acknowledgment of individuals’ rights to autonomy.^3^ Patient participation has centred on patients’ involvement in decision-making about their care, or shared decision-making. Such involvement is attained through dialogue with health-care professionals (HCPs), attuned to the patient preferences, in which patients’ experiential and professionals’ expert knowledge are shared.^4^ Yet patient participation is also defined in a broader sense, including developing relationships, sharing information and decisions and managing self-care.^5–7^ Due to the complexity of the concept, many terms are regarded integral to it, such as involvement, partnership, person-centred care and shared decision-making.^5,7^

According to almost all conceptual papers on patient participation, the impact of participation is invariably positive,^4,6–8^ involving outcomes such as empowerment, better treatment outcomes, increased patient satisfaction and improved quality of care. The results of these conceptual analyses indicate that participation presents itself as ideal and person-centred, as it accords with patients’ needs, values and preferences.^5^ However, it has been reported that the ideal of patient participation can be difficult to attain due to existing barriers such as a lack of time, finance and resources, impractical physical environment and unfavourable leadership and culture.^7,8^ Thus, it is of particular interest that one of the most unexpected outcome of our earlier concept analysis of patient participation, conducted from a person-centred perspective, was that patients do not see all participation as ‘person-centred’.^5^ Rather, they described, in various studies, forms of non-ideal (disrespectful) participation also—perhaps more felicitously categorised as ‘simulacra of participation’ or ‘participation in name only’—characterised by communication struggles between HCPs and patients, where HCPs either do not allow patients the desired kind/extent of participation or force them into unwanted participation. We will refer to such participation as ‘constrained participation.’

The present article constitutes a proof-of-concept study of the real-world traction of a concept of ‘constrained participation,’ straddling the standard distinction between ‘patient participation’ and ‘patient non-participation’ in health care. More specifically, we argue that there is conceptual space not only for a clearly defined concept of ‘constrained participation’ but also for its two sub-concepts, termed ‘fought-for participation’ and ‘forced-to participation,’ which we characterise below.^1^

We are particularly interested in the relevance of the concept of ‘constrained participation’ in the context of the compassionate care of vulnerable older people in general and in geriatric nursing in particular (as indicated in the penultimate section); however, we also draw on other sources as the concept has potential application across wider cohorts of vulnerable patients. We begin in the following section by rehearsing some conceptual findings from our previous *Nursing Ethics* paper on person-centred participation^5^ which already identified a need for the concept in question. We then zoom in more closely, in the third section, on ‘constrained participation’ and its proposed sub-concepts. In the fourth section, we apply some of the already-established considerations to themes in the care of vulnerable older adults. We close by eliciting some characterological, educational and clinical implications of adding these new tools to the conceptual repertoire of nursing practice and education.

One of the most remarkable features of the radical turn towards ideals of patient autonomy and person-centred care, and away from historically entrenched forms of medical paternalism, in the last 50 years or so is that these ideals may seem to be motivated equally by concerns stemming from the three competing ethical theories: Kantian deontology, Millian utilitarianism and Aristotelian virtue ethics. Whereas the first theory grounds person-centred care and patient autonomy in the unconditional requirement of equal respect for all persons as inhabitants in a ‘kingdom of ends,’ utilitarians may argue that allowing patients to participate in, or even have the final say on, decisions relating to their care is, in fact, conducive to the greatest happiness of the greatest number of people/patients. Furthermore, as the third theory is all about the cultivation and exhibition of the individual’s own intellectual virtue of *phronesis* (practical wisdom in moral decision-making), Aristotelians can argue that their theory is actually a powerful wellspring of the ideals in questions also.

That said, historically, the Kantian influence on ideals of patient autonomy and participation has been the strongest; and herein may lie the lack of attention paid to the conceptual ‘grey areas’ between patient participation and non-participation. Because of the formulaic rigidity of the Kantian system of ‘categorical imperatives’, the pendulum has perhaps swung too radically from one extreme to the other, without any sense of the Aristotelian insight that all virtues lie in a golden mean between the extremes of excess and deficiency.^9,10^ Indeed, we aim to demonstrate below that just as strenuously fought-for participation does not count as true patient participation in the context of person-centred care, neither does being forced to participate in processes of decision-making that the patient may, for some reason, prefer to be left out of. To put this in characterological terms, the moral and intellectual virtues of HCPs and patients find their optimal actualisation in a reflective equilibrium between paternalistic guidance/advice and autonomous uptake/decision-making. Good qualities–not only the correctives of bad ones–call for medial measures; and this insight needs to find its way into the education of compassionate and caring HCPs.^2^

## Revisiting patients’ perspectives on person-centred participation

Our framework analysis^5^ based on an integrative review of 60 qualitative studies of patients’ own perspectives of ideal patient participation, has garnered a significant number of academic citations, which shows that there is an appetite for a deeper conceptual understanding of issues that relate to different facets of patient participation. The selected studies reflected considerable geographical and cultural diversity since 39 studies came from Europe, 16 from North America, four from Australia and New Zealand and one from Asia.

As patients understand these concepts, ‘person-centred care’ is tantamount to ‘ideal patient participation’ and is based on patients’ experiences, values, preferences and needs.^5^ On a virtue ethical reading, such participation can be viewed as the golden mean of participation that lies between the extremes of excess and deficiency.^10^ By this we do not mean that patient participation itself constitutes a virtue. However, we consider it to be both a moral and an executive virtue of HCPs to provide patients with the proper scope to exercise their autonomy. Indeed, insofar as the concern with patient participatory autonomy can be grounded in a virtue ethical framework rather than a deontological one, as suggested above, this virtue may be seen to fall within the rubric of HCPs’ professional *phronesis*.^11^ Notably, our earlier analysis of patient participation was the first one explicitly conducted from a person-centred perspective. Yet subsequent analyses of the concept emphasise a person-centred approach,^4,7,8^ for example via respect for patients’ experiences and preferences, more than earlier analyses of the concept did.^6,12^

**Figure 1.**
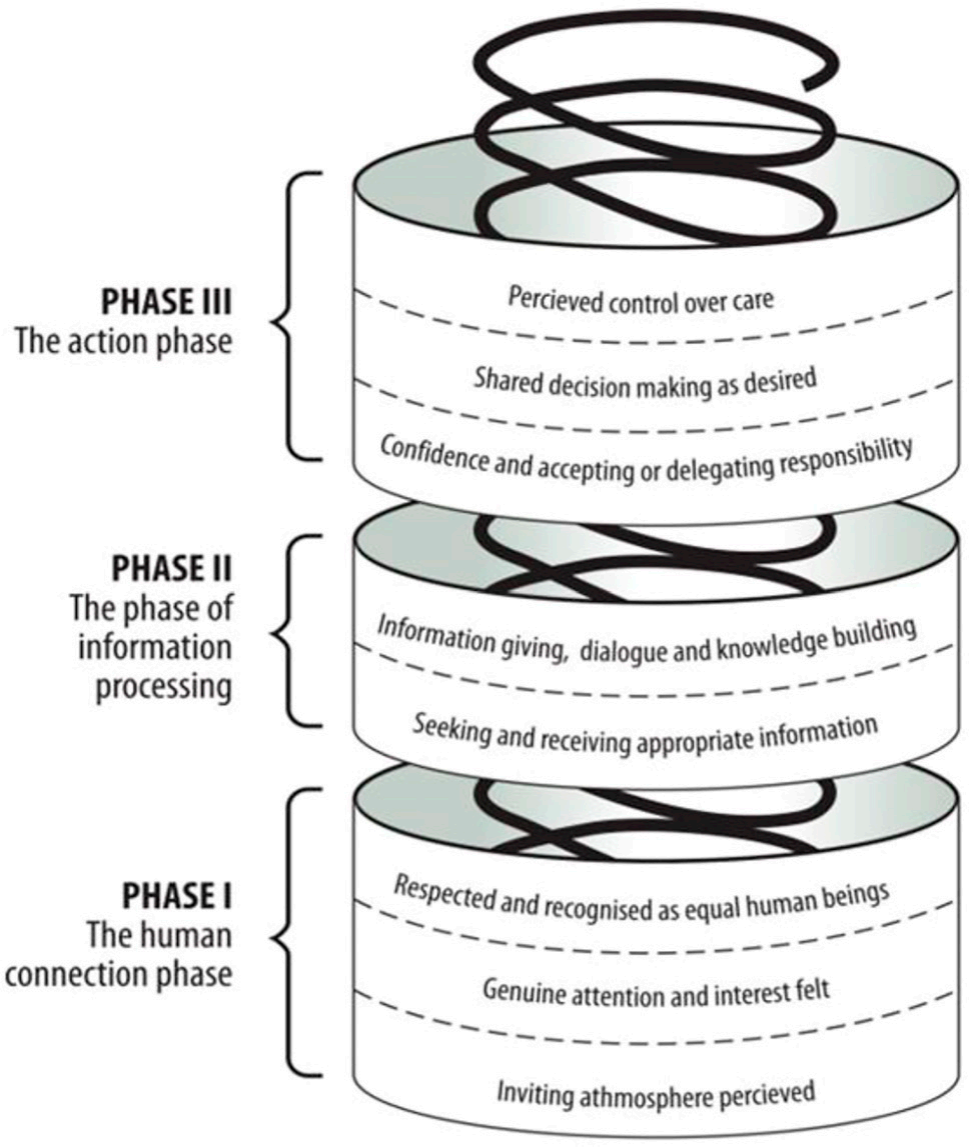
Conceptual framework of person-centred participation in health care.^5^

In our above analysis, the following terms were regarded as integral to patient participation: ‘involvement,’ ‘partnership,’ ‘decision-making,’ ‘shared decision-making,’ ‘patient/person/client-centredness’ and ‘patient/person/client care/practice’. In all the 60 studies, patients’ experiences of patient participation involved direct communication with HCPs regarding their own health care. Those studies were then synthesised, which resulted in the identification of three intertwined phases of ideal participation or ‘person-centred participation’ and offered the conceptual framework depicted in Figure 1.

In the first phase, which provides a grounding for the other phases, a human connection between patients and HCPs is developed, within which patients perceive respect and recognition as persons and as equal human beings. Perception of an inviting atmosphere, such as friendly HCPs, respect in the health-care ethos and good access to health care services, sets the initial stage of this phase. In the second stage, patients experience that they are shown genuine interest and attention, to which many communicational features are integral, such as active listening, invitation to speak and patients being taken seriously HCPs. Those features are antecedents of third stage in which the human connection is strengthened by respect for and recognition of patients’ equality and their unique and holistic properties in interaction between them and HCPs. Here, patients expressed the importance of being respected and viewed from a holistic perspective regardless of whether they were asked about their experience of patient participation, person-centred care, partnership or involvement in shared decision-making.

In some cases, patient participation is confined only to the human-connection phase as a whole or even only to one of its stages or sub-stages. Similarly, if patients so choose or their condition allows, their participation advances further into and merges with each of the other phases, within which it can be confined to only one or all of their stages and sub-stages.

In the second phase of ideal participation, that of information processing, patients exchange information with HCPs. In the beginning of this phase, patients seek and receive appropriate information as preferred. As the second phase progresses, there is a need for deep understanding of the health condition, developed by ongoing dialogue with HCPs and knowledge-building which ideally is a prerequisite for decision-making.

In the last phase, patients take action towards health problems. It is initiated by their accepting/delegating responsibility and progressing into making final decisions regarding the selection of treatment choices in accordance with their preferences. In the final stage of this phase, patients perceive control over their own care by conforming to care according to their wishes, or managing care by carrying out care activities according to their preferences. We hypothesised that the lacuna in previous analyses, mentioned at the outset (of not identifying cases of non-person-centred participation) could be traced to the fact that patients have not typically been asked questions that could identify non-ideal participation, such as ‘Have you participated less/more in your healthcare than you would have preferred?’ (5). However, studies show that patient preferences for participation differ between cultures,^13^ individuals^14^ and through each individual’s phases of illness.^15^ Moreover, a mismatch between patient preferences for participation and the actual participation is linked to negative patient experiences, such as frustrations,^16^ burden,^17^ and dissatisfaction with care.^13^ This is at odds with other conceptual studies that, as already outlined, addressed the positive impact of patient participation.^4,6–8^

It can be argued that this discrepancy it due to the fact that these conceptual papers have not differentiated between person-centred participation *qua* preference-based versus non-preference-based participation. However, Eldh et al.^14^ clearly differentiate between these facets of patient participation in their paper about a new tool to measure patient preferences for participation. In their paper, they designate ‘good’ patient participation as preference-based participation, which in our terms is ‘person-centred participation.’ Participation that is non-preference based is referred to them simply as ‘bad’ participation.

While Eldh et al.’s^14^ construct of ‘bad participation’ is consistent with the anomalous finding of our earlier analysis, it is somewhat lacking in conceptual nuance. In our analysis^5^ we had space only to register the anomalous finding briefly and offer hints about how it could be explained. However, this phenomenon of what we have referred to as ‘constrained participation’ has continued to intrigue us because, despite more examples in subsequent empirical papers, it has yet to emerge as a theme in overview articles and integrative reviews.

In order to set ‘constrained participation’ in an historical and philosophical context, we begin the following section with a brief review of some more general conceptual considerations before illustrating the ‘new’ concept and its two sub-concepts with some examples from the relevant literature. We acknowledge the need for a similar integrative review of studies relevant to the concept of ‘constrained participation’ as we did earlier for ‘person-centred participation’,^5^ and we aim for such a review in the future. However, at this early juncture, we deem it important to introduce the construct through a simpler exploratory proof-of-concept (PoC) study. Methodologically speaking, a PoC study introduces a new construct or idea and argues, through preliminary empirical evidence, that it is potentially operationalisable; has real-world traction; and may serve to complement or finesse a given theoretical or conceptual model. Epistemologically, the PoC method challenges theory-first approaches that consider any given conceptual repertoire to be prefigured by theory.^18^ Rather, the PoC method suggests that any theoretical model in a field such as nursing is potentially revisable in light of new constructs that fit with subjects’ actual experiences.

In the field of concept analyses, a proof-of-concept study stands in similar relationship to a full-blown concept analysis (or a framework analysis, like we conducted)^5^ as an empirical pilot study stands to an empirical main study.^18^ Before conducting a full analysis of a new concept, it is helpful to gather evidence about whether there is potential space for it on the conceptual map of surrounding and already-existing concepts. As about 10 of the 60 articles we had already scrutinised in our comprehensive 2014 framework analysis of ‘patient participation’^5^ had turned up the anomalous notion of relationships that are more felicitously described as ‘participation’ than ‘non-participation’ but are still constrained, we decided to conduct a preliminary search of post-2014 articles to explore whether the same phenomena emerged there. We continued to search until we reached what we deemed to be a saturation point; and we also ascertained that the concept of ‘constrained participation’ (or either of its apparent sub-concepts) had still not registered on the radar of any integrative articles in this field. Hence, the need for the present proof-of-concept study. However, as indicated above, we plan to follow up this preliminary study ourselves with a comprehensive framework analysis of ‘constrained participation’, and we also encourage other researchers to help ‘join the conceptual dots’ that we have discovered and describe below.

## ‘Constrained participation’ and its two sub-concepts

Paternalism^3^ used to be the reigning model of decision-making in the medical and more general healthcare/care-home spheres. However, the last half a century has witnessed a serious backlash against such paternalism as disrespectful of personhood (on a Kantian view), inimical to overall well-being (on a utilitarian view) and non-virtuous (on a virtue ethical view). Practical concerns have also played their part: for example, HCPs’ heightened fear of litigation if they ignore patients’ wishes, withhold the truth from them or do not obtain a formal and explicit ‘informed consent’ prior to clinical decisions.

Nowadays, autonomy and paternalism are often presented as binaries: two incompatible models for healthcare. This depiction overlooks the fact that these two are treacherous as binaries, often proposing to usurp one another. For instance, there are many different forms of paternalism, hard versus soft, broad versus narrow, pure versus impure,^19^ each with its own distinctive set of assumptions. Second, paternalism and autonomy often operate fairly successfully side by side, depending on various individual and contextual care factors.^20^ Third, the ideal of autonomy comes with a well-known set of paradoxes, many of which can be traced back to Rousseau’s view of so-called positive liberty in his *Social Contract*.^21^ In that work Rousseau argued that because the general public did not understand the nature or value of their own autonomy and freedom, authorities would need to ‘force them to be free’. While Rousseau’s work represents a somewhat extreme version of this paradox, it presumably lurks in more practical healthcare settings also. HCPs may thus be so committed to the ideals of anti-paternalism and of patient autonomy that they revert to paternalistic measures to force patients to exercise their autonomy. The result is what we call ‘forced-to participation’ and identify as one of the two sub-concepts of ‘constrained participation’ below.

Prior to that, it is instructive to explore another, and perhaps more common, variant of what patients seem to identify as non-ideal participation. That variant comprises instances of participation which does take place but is ‘constrained’ in the sense of having been painfully fought for and/or not eventually assuming the precise incarnations that the patient would prefer. ‘Fought-for participation’ of this kind can assume various forms. Many examples of such participation were found in the literature: examples which can be contrasted with ideal participation occurring at all the phases of patient participation. We offer some brief illustrations below at each of the phases to validate the concept.

Patients may perceive themselves as having had to fight for receiving attention (i.e., for being seen and heard, taken seriously, allowed to express their symptoms). For example, in a study involving video-recorded consultations with physiotherapists in Hong Kong, patients’ own initiations of symptom-talk were scantily and belatedly acknowledged.^22^ Another study showed how elderly patients had developed strategies to connect with HCPs which they claimed were not responsive due to the stressful environment in the hospital ward.^23^ Moreover, patients may struggle to have their knowledge respected. The results of the study of Abhyankar et al.^24^ which explored women’s experiences of receiving care for pelvic organ prolapse, provide an example of such struggle. They showed that the women had to fight to have their embodied knowledge of the first symptoms of the prolapse, such as co-existing urinary leakage, respected by their GP. This tended to result in a lack of early diagnosis or treatment such as pelvic exercises. In many cases, the women’s knowledge was first respected when the prolapse had progressed to the extent that surgery was needed. These three examples of patients striving for connection and respect form HCPs can be viewed as deficiencies of the golden mean of the human connection developed in the first phase of person-centred participation.^5^

Patients sometimes have to fight for receiving information, which we regard as a deficiency within the phase of information processing of ideal participation in which patients receive information tailored to their individual needs. For example, a study involving older patients’ perceptions of dialysis initiation illustrated unsuccessful and frustrating efforts by many of these patients in obtaining important information from HCPs about their prognosis after the dialysis initiation.^25^

Examples of patients striving to influence care decisions or gain control over care can be regarded as deficiencies in the action phase of ideal participation in which HCPs facilitate such influences and control.^5^ A multiple-case study exploring experiences of elderly people in hospital and their relatives provides a distinct example of when patients are not allowed to be involved in decision-making in the way they would prefer.^16^ One case involved an elderly woman who described her several futile attempts to reach out to HCPs for participating in decision-making about her major concern of potentially having to move from her home and find new future home arrangements due to her recent functional decline. Another study illuminated how elderly recipients of home care struggled for being in control of their care by continuously being on the guard in ‘supervising’ care givers in the task of providing care as they wished.^26^

A common characteristic of the problematic participatory processes illuminated above is that the professionals in question explicitly claim to favour an empowering person-centred approach to participation; yet in reality (or at least as perceived by patients), they do not practise what they preach (see e.g. Yip and Schoeb).^22^ Preferences by patients about procedures such as shift-to-shift nursing handovers are conveniently overlooked if they conflict with standard practices,^27^ as long as they patients do not complain overtly about the nature of the (presumably) participatory processes. All in all, what characterises ‘fought-for participation’ seems to be a subtle abuse of power where protocols about informed consent and person-centred participation may be followed in letter—but definitely not in spirit.

Let us now turn to the second sub-concept of ‘constrained participation’, namely ‘forced-to participation,’ which is more philosophically intriguing but also more difficult to identify properly, as the ideal of patient participation seems to be followed not only in letter but also in spirit. The only problem is that the ‘spirit’ is taken to such an extreme that the end-product becomes a perversion of the original ideal. We here enter the thorny territory of ‘paternalistic anti-paternalism’. Once again, ‘forced-to participation’ can assume many forms, although all of them involve HCPs imposing participatory processes on patients, typically in the name of ‘patient autonomy’, but where patients would either rather be left to their own devices or simply having the burden of complex decision-making, care responsibilities and management shifted away from them. Accordingly, we view ‘forced-to participation’—as in the below examples—in virtue ethical terms as constituting excesses in the action phase of person-centred participation in which the extent of active participation should ideally be in line with patients’ preferences, involving delegation of decision-making and care responsibilities. Notably, registered examples of excesses of ideal participation were limited to the action phase.

Holdsworth et al.^15^ provide an example of such excesses of ‘forced-to participation’ in their study involving cancer patients’ perception of decision-making within which they were forced to take decisions about issues that they feel unqualified to deal with. The results showed that shared decision-making was uncomfortable for those patients, who preferred that a physician led the decision and who had difficulties in assessing the information presented to them. Another study illustrates how patients are forced to take responsibility for processes for which they would rather not be held accountable.^17^ It shows how patients with end-stage kidney disease who were on hospital haemodialysis were forced to take more responsibility than they preferred for coordinating their transition between the haemodialysis ward and other health services. The patients explained that they needed to do so because of collaborative deficiencies of HCPs in these health sectors, which hampered their overall care.

Moreover, patients are sometimes required to co-operate in and comply with regimens, involving performing activities that they find difficult to manage physically or mentally on their own without professional support as preferred. Such constrained participation was for example illustrated in a study in which patients’ participation in a fast-track colonic surgery was explored.^28^ These patients strove to live up to HCPs’ expectations by adapting to the post-operative regimen unconditionally and complying with the role of being ‘good’ and ‘cooperative’. They did so in spite of unpleasant reactions such as nausea or pain occurring, which made them feel weak and incompetent regarding actively behaving as recommended. Their role of being a ‘good’ and ‘obedient’ patient was dominant. Instead of ideal participation, the asymmetric power relationship between the patients and professionals was perpetuated through a forced-to process, impeding the patients’ well-being.

We must admit that we have been more surprised about the apparent prevalence of ‘forced-to participation’ than ‘fought-for participation’, as the former seems to contradict some received wisdoms about the popularity and topicality of anti-paternalism in healthcare ethics.^4^ Why is it that the laudable ideals of patient autonomy and informed consent have become a poisoned chalice for some patients? This question calls for an extended historical and philosophical discussion that would mostly be outside of our present remit. We simply wish to flag a few possible considerations.

First, partly through a deontological focus on patients’ rights, partly through a legalistic demand for explicit participation, securing informed consent seems to have become a formalistic tick-box exercise, which is no longer geared towards patients’ overall welfare.^11^

Second, these two demands have evoked a ‘Hedgehog mentality’ among HCPs where ‘one big idea’ (here: patients’ autonomy) becomes something of an obsession, without sufficient attention being paid to situational complexities.^29^ This leads to patients being treated as one homogeneous group, rather than a collection of heterogeneous individuals with different needs, wants and preferences.^30^

Third, this tendency towards homogeneity has, in turn, motivated disengagement with values that were originally meant to mitigate the increased focus on patient autonomy, such as ‘appropriate engagement’ rather than forced engagement, and ‘intersectionality’: namely, seeing patients as made up of various intersecting characteristics having to do with their age, gender, upbringing, education, life experiences, health status, etc., and being treated accordingly.^31^

As important as the ideal of patient autonomy is, it must not be fetishised and turned against itself so that its anti-paternalism itself becomes paternalistic (see various articles in Cooke and Kothari).^32^ What needs to be remembered is that it is no less paternalistic to force patients to participate in processes from which they would prefer to be left out of than it is to debar them from participating in processes in which they would prefer to be involved.

To close this section, we wish to offer a methodological word of caution that mirrors our earlier misgiving about seeing autonomy and paternalism as strict binaries. ‘Autonomy’ is an open-textured naturalistic concept (similar to concepts such as ‘freedom’ and ‘power’), as distinct from the closed concepts of logic and mathematics.^33^ Hence, there is no ideal ‘pure’ form of autonomy in which an individual exercises choices in a vacuum, fully unencumbered by the influence of others. In that sense, ideal (autonomous) participation versus constrained (non-autonomous) participation are better seen as different rungs in a ladder, leading from constraint to freedom, rather than as binary concepts. For researchers exploring ‘constrained participation’ in its two incarnations, described above, it is thus important not to over-interpret findings of all less-than-ideal participation as necessarily falling into the categories of forced-to or fought-for participation. In our literature search, we were careful not to categorise instances of perceived non-ideal participation as constrained, in either of its two proposed senses, unless it clearly transpired from patients’ own words that they experienced frustration and an explicit sense of disengagement and disempowerment with respect to the participation offered by the relevant HCPs.

## ‘Constrained participation’ in the context of caring for vulnerable older patients

We have noticed that many of the paradigmatic examples of ‘constrained participation’—of both sub-varieties—found in the literature stem from the context of caring for vulnerable *older* patients. That is perhaps not surprising as this group possesses a number of characteristics that may predispose it to both ‘fought-for’ and ‘forced-to’ participation. For example, regarding the first variety, the history of the care of older people unfortunately carries many examples of older people being infantilised and patronised;^34^ hence those who have a mind of their own may have to fight hard for having their voices heard. Regarding the second variety, many in this group were brought up in an era when medical paternalism was the norm; hence they may have been habituated into wanting to defer to the person in authority (doctor, nurse, carer) rather than making autonomous decisions.

Globally, the number and proportion of old people in the population is increasing rapidly and will accelerate in the next decades.^35^ With increasing age, functional decline and chronic illness, including cognitive impairment, become more prevalent^36^ which increases the needs for health care.^37^ Being dependent on care threatens autonomy and self-determination.^34,38^ Thus, it is important to secure a deeper understanding of what making ‘independent choices, without domination or suppression’,^39^ means for this cohort. It is already clear from the literature canvassed above that many HCPs possess a very narrow understanding of ‘domination and suppression’, where those terms refer solely to deliberate, active interferences with the ability to make autonomous choices. However, this means that more subtle cases of non-ideal participation are negligently missed or cavalierly ignored. This will be particularly true in contexts such as geriatric wards, residential homes and nursing homes where the distinction between autonomy and paternalism is already quite blurred and these two modes of care can, at the best of times, operate fairly unproblematically side by side.^20^ However, paternalism inhibiting participation in autonomous decision in home care settings is prevalent.^38^

The most worrisome sub-group in this regard comprises older patients at various stages of cognitive impairment: patients who are particularly vulnerable to violations of rights and, in real if rare cases, to outright ‘elder abuse’.^29^ It is well known that cognitive impairment can be very context-specific. A person incapable of making autonomous choices about some facets of her life may still be competent enough—and have a desire—to adjudicate on other areas of her care and day routine. It is important, therefore, that nurses and carers remain alert to the patient’s own wishes and honour those as far as possible,^31^ rather than simply doing what they think is best for the patient in the name of ‘compassionate caring’.

A recent integrative review of older people’s perceived autonomy in residential care has, by collating a host of specific findings from different contexts, shed new light on some of the issues involved in securing ideal participation for this group.^34^ What transpired from their study is, inter alia, that autonomous decision-making is positively related to subjective health and the absence of stress, depression and apathy. Although this review did not positively identify the two sub-concepts that we have tried to validate in this article, it revealed that, in some cases, the residents valued relatedness (to HCPs) over autonomy and were willing to put up with gentle forms of coercion, provided that full trust had been established with the HCPs in question, who ‘knew what was best for them’.^34^ This finding relates potentially to the ‘forced-to participation’ sub-concept in the sense that compelling old people to take decisions about themselves in situations where they would prefer to defer to ‘an expert’ may undermine trust rather than facilitating it.

No serious theorist would reject the claim that health and care services should ‘aim to preserve dignity and autonomy and minimise stress’ among vulnerable older patients.^30^ The question is simply how this is best achieved; and in this article we have focused on cases where the ideals in question are not actualised although some sort of non-ideal ‘participation’ does eventually materialise. One further thing to note in this regard is that many older people are very protective of their boundaries,^31^ being brought up in an era before social-media fuelled self-exposures became the norm. To understand those boundaries, HCPs need to understand the person and establish a rapport with her, which can in some cases be both time-consuming and taxing.^39,40^

## Some characterological, educational and clinical implications

A large study of nurses in the UK revealed that, contrary to other professions studied, nurses’ reliance on their own moral compass and practical wisdom decreased with work experience, whereas their reliance on formal codes and rules increased. This was interpreted as being indicative of a profession in a potential moral crisis and prone to burn-out.^41^ We hazard to hypothesise that the somewhat formulaic and mechanical understanding of autonomy, which possibly gives rise to both ‘forced-to’ and ‘fought-for’ participation, may have its roots in a deeper malaise affecting the nursing profession. This makes it difficult to suggest correctives that are limited only to ameliorating the distortions of ideal patient participation unearthed in this article.

With respect to the 6Cs highlighted in the 2012 NHS England vision and strategy for nursing and care staff,^42^ the C of *communication* stands out for present purposes.^5^ Both ‘fought-for’ and ‘forced-to’ participation seem to indicate a breakdown in communication between HCPs and patients and, more specifically, the lack of what Vanlaere and Gastmans^43^ call ‘critical companionship’ in nursing. This critical companionship involves both criticality towards written codes, colleagues, nursing theories and, indeed, patients – where no claim is taken at face value without interrogating it critically. Most importantly, ‘critical companionship’ involves criticality towards oneself, where one reflects upon and questions one’s own practices. This stance thus overlaps considerably with the time-honoured intellectual virtue of *phronesis* or practical wisdom.^44^ ‘Critical companionship’ also involves the capacity for ‘real dialogues’ with patients about their wishes: dialogues that do not always necessarily need to be uncritical and non-confrontational, although they must always remain considerate.^40,45^

Notably, often the dialogue is of more importance to patients than the decision as such.^5^ A recent synthesis of shared decision-making in line with the person-centred approach illuminates that gaining understanding of the patients’ preferences and wishes is central for older people with complex health needs.^39^ Furthermore, communication skills of HCPs and relationship-building are essential for eliciting those preferences and responding to, as well as guiding, patients in the shared decision-making process. The results of the synthesis agree with a virtue ethical analysis of patient participation in terms of a golden mean, as suggested above.

The principles of autonomy and ideal patient participation must be taught with much more nuance in nursing education than seems to be the case nowadays in many nursing departments. It needs to be conveyed clearly to students that although informed consent was, for instance, a principle well-designed to deal with problems of pervasive paternalism in healthcare, it is not a silver bullet that can be used to mindlessly kill off all traces of paternalistic interventions. It is as much, or even more, the duty of the HCP to help patients overcome some of their cognitive biases (e.g., by teaching them techniques to avoid such biases) as it is to elicit from them an approval of some ready-made treatment plan. The precursor to a well-informed autonomous decision-making is thus often what Levy^45^ calls ‘confrontational counselling’ rather than simply forcing patients to listen inactively to some (possibly unwanted) information and signing on a dotted line. Students must be taught that true autonomy can only be exhibited under conditions that enable patients to be motivated by goals with which they appropriately identity. This means, for example, that the patients must not be in a condition of undue stress or fatigue^45^.^6^ Forcing patients to participate under such conditions is a classic example of what we have called ‘forced-to participation’: a far cry from any participation that deserves the name ‘ideal.’

All in all, the takeaway lesson for students must be that ideal patient participation can only take place in a ‘goldilocks zone’ between excess and deficiency. The educational focus has so far been on the deficiency side, but it must include the excess side also. So, while we welcome Eldh et al.’s^14^ earlier-mentioned research tool to distinguish between what they call simply ‘bad’ and ‘good’ patient participation, we agree with their conclusion that for a ‘thick concept’ like patient participation, ‘the complexity is more pronounced’. Our new conceptualisation adds a new layer of complexity that will help nursing students better grasp the multi-dimensionality of patient participation.

From a clinical perspective, our ‘proof of concept’ of the two varieties of ‘constrained participation’ carries some further implications. By appreciating patients’ fight for participation at the initial phases of participation, involving their strivings for receiving attention, respect and information from HCPs, nurses can become more aware of and responsive to the struggles patients sometimes have to face when initiating their participation. Similarly, nurses’ awareness can be raised by illustrating examples of both fought-for or forced-to participation, in the advanced stages of patient participation, either when those involve patients’ frustrated attempts to influence care decisions or gain control over care as they prefer or, on the other hand, cases of burdensome decision-making and care responsibilities being forced upon them.

Mindful that what one patient may experience as ideal participation, another patient may experience as fought-for or forced-to participation, our conclusion is that clinical assessment of patient preferences for participation is of paramount importance, in particular in caring for old people with complex needs. Such assessment can proceed into reflective dialogue with patients on their needs and critical reflection upon one’s own professional practice, which may in favourable situations advance into true *phronesis*. Arguably, such *phronetic* practice can optimise the acknowledged positive impact of patient participation, such as empowerment, patient satisfaction, patient safety, as well as improved quality and outcome of care.

## Notes

1 While the first sub-concept may be deemed more salient in terms of patient advocacy, the second carries significant philosophical significance as it harks back to the Rousseauian notion of being ‘forced to be free’ and, by implication, to lingering paradoxes about the idea of heteronomously secured autonomy.

2 While most of the research drawn upon for our present proof-of-concept study features nurse–patient relationships, we also consulted research findings about other cohorts of HCPs insofar as those findings indicated compromised forms of patient participation.

3 Paternalism is the view that A has the right to force x upon B in a way that interferes with B’s autonomy and freedom, without B’s consent, for the reason that x is in B’s best interest.^19^

4 In his contribution to the current Special Issue, Paul Snelling draws an intriguing parallel between the nurse–patient and teacher–student relationships, which is particularly pertinent if the teacher is also a registered nurse. We can think of various examples of (nursing) students being subjected to either fought-for or forced-to participation in their studies, although exploring such occurrences is outside the purview of the present article.

5 The other Cs are care, compassion, competence, courage and commitment. All the Cs could potentially be related to the topic of this article, although we single out communication for attention here. Obviously, the 6Cs are also variously inter-related, for example ‘active listening’ is both part of good communication and compassionate care.^42^

6 Levy^45^ correctly points out that reasonable misgivings about the limitations of patient autonomy, such as those caused by the various fallibilities and biases of human reason, or psychological obstacles to its proper functioning, are often wrongly cast as reactionary and corrosive of the ideal of patient autonomy.
